# Pulsed Electric Field Treatment Promotes Lipid Extraction on Fresh Oleaginous Yeast *Saitozyma podzolica* DSM 27192

**DOI:** 10.3389/fbioe.2020.575379

**Published:** 2020-09-11

**Authors:** Olga Gorte, Natalja Nazarova, Ioannis Papachristou, Rüdiger Wüstner, Klaus Leber, Christoph Syldatk, Katrin Ochsenreither, Wolfgang Frey, Aude Silve

**Affiliations:** ^1^Institute of Process Engineering in Life Science 2: Technical Biology, Karlsruhe Institute of Technology, Karlsruhe, Germany; ^2^Institute for Pulse Power and Microwave Technology (IHM), Karlsruhe Institute of Technology, Karlsruhe, Germany

**Keywords:** oleaginous yeast, pulsed electric field, pulsed electric field assisted extraction, electroporation, lipid, solvent extraction, fresh biomass

## Abstract

This study reports on the use of pulsed electric field (PEF) as a pre-treatment step to enhance lipid extraction yield using extraction with ethanol-hexane blend on fresh oleaginous yeast *Saitozyma podzolica.* The yeasts were cultivated on nitrogen-depleted condition and had a lipid content of 26.4 ± 4.6% of dry weight. PEF-treatment was applied on the yeast suspension either directly after harvesting (unwashed route) or after a washing step (washed route) which induced a reduction of conductivity by a factor eight. In both cases, cell concentration was 20 g of biomass per liter of suspension. In the unwashed route, the lipid extraction efficiency increased from 7% (untreated) to 54% thanks to PEF-treatment. In case an additional washing step was added after PEF-treatment, up to 81% of the lipid content could be recovered. The washed route was even more efficient since lipid extraction yields increased from 26% (untreated) to 99% of total lipid. The energy input for the PEF-treatment never exceeded 150 kJ per liter of initial suspension. The best lipid recovery scenario was obtained using pulses of 1 μs, an electric field of 40 kV/cm and it required slightly less than 11 MJ/kg_LIPID_. This amount of energy can be further reduced by at least a factor five by optimizing the treatment and especially by increasing the concentration of the treated biomass. The process can be easily up-scaled and does not require any expensive handling of the biomass such as freezing or freeze-drying. These findings demonstrate the potential benefit of PEF-treatment in the downstream processing of oleaginous yeast. From a basic research point of view, the influence of conductivity on PEF energy requirements and extraction yields was examined, and results suggest a higher efficiency of PEF-treatment in terms of energy when treatment is performed at lower conductivity.

## Graphical Abstract

Pulsed electric field treatment performed on fresh yeast biomass induced permeabilisation of cells and greatly improved the yields of subsequent lipid extraction using ethanol and hexane.

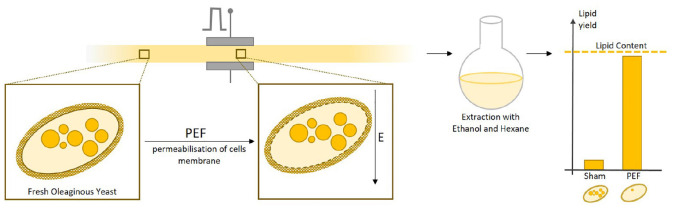

## Introduction

Pending population growth and the associated energy and resources demand bear decisive economical and societal challenges. The foreseeable depletion of crude oil (Vasudevan and Briggs, 2008) and the urgent need for the reduction of greenhouse gas emissions to prevent further climate change, highlight the need for sustainable alternatives. Using oleaginous plants as alternative source of oil raises the competition between food and raw materials production, since agricultural land is limited (Lee and Lavoie, 2013). Into the bargain, an expansion of oil seed cultures would lead to forest land destruction (Escobar et al., 2009). Microbial oils, known as single cell oils (SCO), produced by yeast, microalgae, fungi, and bacteria may overcome all these challenges and act as potential feedstock for crude and plant oil for various applications such as fuels, additives for food and cosmetics, and building blocks for oleochemicals (Ochsenreither et al., 2016; Probst et al., 2016; Vasconcelos et al., 2019).

One of the most promising oleaginous microorganisms are yeasts, since they are able to accumulate more than 70% of their cell dry weight (CDW) of lipids (Ratledge, 1991; Probst et al., 2016). The production of SCO by yeasts is independent of season, climate, and location and requires limited amounts of area for cultivation. Additionally, high growth rate and oil productivity are achieved due to short duplication time and the possibility to up-scale cultivation processes, which renders an industrial use realistic (Li et al., 2008; Ageitos et al., 2011). Furthermore, unconventional carbon sources, e.g., lignocellulosic material and waste from food and other industries can be metabolized, enabling waste recycling and guaranteeing a sustainable process (Ward and Singh, 2005; Li et al., 2008; Ling et al., 2013; Liu et al., 2015; Fei et al., 2016; Kot et al., 2017). In the event of carbon excess and nutrient limitation, e.g., nitrogen or phosphate, SCOs are produced as intracellular storage lipids, mainly in the form of neutral triacylglycerols (TAGs) (Ratledge, 2004).

Despite all these advantages, production of SCO resembling plant oils is currently too expensive to allow commercialization (Ratledge and Cohen, 2008; Leong et al., 2018). One of the major challenges for the industrialization is the development of an efficient, cost-effective and low energy consuming method for the recovery of the intracellular product. The rigid and robust properties of the yeast cell wall significantly contribute to poor SCO accessibility and resistance against organic solvents (Jacob, 1992). For a successful oil extraction, cell pre-treatment is pivotal. However, a universally accepted SCO recovery method does not exist and the most advantageous method needs to be identified individually for each species. In literature a number of different techniques, diverse in nature such as mechanical [high-pressure homogenization (Thiru et al., 2011) and solid shear by bead milling (Meullemiestre et al., 2016; Vasconcelos et al., 2018)], physical [microwave (Yu et al., 2015; Meullemiestre et al., 2016) and sonication (Zhang et al., 2014)], chemical [organic solvents (Breil et al., 2016) and acidic hydrolysis (Yu et al., 2015)] and biological [enzymatic lysis (Jin et al., 2012)] have been applied successfully for the extraction of various yeasts’ oils. Most methods lack energy-efficiency or up-scalability.

In this context, we have recently evaluated industrially relevant cell disruption methods on laboratory scale using frozen and freeze-dried biomass of the unconventional yeast *Saitozyma podzolica* DSM 27192 (Gorte et al., 2020). With regard to energy consumption, the direct transesterification of freeze-dried biomass to fatty acid methyl esters (FAMEs) for biodiesel production turned out to be the most energy consuming technique with 5588 kWh/kg FAME ≈ 20116.8 MJ/kg FAME. This method can therefore only be considered as an analytical technique. Other tested lipid recovery methods were high pressure homogenization (95 kWh/kg lipid = 342 MJ/kg lipid) and bead milling (96 kWh/kg lipid = 345.6 MJ/kg lipid) of frozen biomass prior to mixed solvent extraction (Gorte et al., 2020). Although latter pre-treatment methods consumed several orders of magnitudes less energy than the first one, they remain too energy consuming. Meullemiestre et al. (2016) revealed similar findings in respect of energy consumption for *Yarrowia lipolytica*, declaring freeze-drying as the most energy consuming pre-treatment technique and bead milling as the least energy demanding. For industrial use, dehydration of biomass before lipid extraction is un-economically due to high costs and energy demand. Therefore, an extraction method which is scalable and efficient on wet biomass, needs to be found (Willis et al., 2014).

Pulsed electric field (PEF) treatment is an alternative pre-treatment method, which relies on the application of short intense electric pulses, that when delivered to biological cells, induce an increase in transmembrane voltage and subsequently an increase in the permeability of the membrane (Teissie et al., 2005; Pakhomov et al., 2010). This process is effective both on animal and plant cells, which has resulted in applications in the medical domain as well as in the food industries. Additionally, this treatment is very efficient on single cells in suspension, which is used for bacteria transfection, a technique routinely employed in molecular biology, but also efficient on complex cell tissue, as implemented in tumor treatment with electro-chemotherapy (Gothelf et al., 2003; Mir, 2006; Mir et al., 2006). In food industry, permeabilization of membranes with PEF is shown to be very effective in extraction processes (Toepfl et al., 2006; Sack and Muller, 2008; Donsì et al., 2010; Sack, 2012; Barba et al., 2015; Raso et al., 2016) e.g., in apple juice production, and some facilities already operate at industrial scale. PEF-treatment has also established itself in other applications such as the processing of potatoes in the snack food industry (Toepfl et al., 2006; Barba et al., 2015). With regard to the extraction of oil from oleaginous biomass, trials have been reported on rapeseed, linseed, olive paste and black cumin seed with results indicating that pre-treatment with PEF can increase lipid extraction yields of traditional pressing methods without impacting quality of the oil (Guderjan et al., 2005; Savova et al., 2005; Puértolas and de Marañón, 2015; Sarkis et al., 2015a,b; Bakhshabadi et al., 2018). More recently, experiments from our laboratory on the microalgae of the species *Auxenochlorella protothecoides*, have shown that PEF-treatment allowed to reach up to 97% of lipid extraction yield after solvent extraction against only 10% in the absence of pre-treatment (Silve et al., 2018b). The energy levels implemented for microalgae are not more than 1.50 MJ per kilogram of microalgae dry weight (DW) and may even be reduced to less than 250 kJ per kilogram DW under certain conditions (Silve et al., 2018a). From other work, it is also known, that PEF-treatment is efficient on several types of yeasts (Aronsson et al., 2005; Suga and Hatakeyama, 2009). The permeabilization induced by PEF-treatment was successfully used for transfection, but also for electro-extraction of molecules, especially of enzymes (Ganeva and Galutzov, 1999; Ganeva et al., 2001). Additionally, it was shown on *Saccharomyces cerevisiae* that PEF-treatment could accelerate autolysis processes with benefits for wine production (Martínez et al., 2016, 2019). Several evidence point at an effect not only on the cell membrane but also on the cell wall, probably as a consequence of the initial permeabilization (Ganeva et al., 1995, 2014).

The present study aims to test whether PEF-treatment applied on an oleaginous yeast can improve subsequent organic solvent-based lipid extraction at reasonable energy consumption. For that purpose, the previous protocols developed for microalgae (Silve et al., 2018a,b) were transposed to the recently characterized oleaginous yeast *Saitozyma podzolica* DSM 27192 (Schulze et al., 2014).

## Materials and Methods

### Microorganism

All experiments were performed with the oleaginous yeasts *Saitozyma podzolica* DSM 27192, which was newly screened from peat bog soil and deposited at the DSMZ culture collection (Deutsche Sammlung von Mikroorganismen und Zellkulturen; Braunschweig; Germany) as *Cryptococcus podzolicus* DSM 27192 by Schulze et al. (2014). After genome sequencing and annotation, the strain was phylogenetically reclassified as *S. podzolica* DSM 27192 (Aliyu et al., 2019).

### Yeast Cultivation

The oleaginous yeast *S. podzolica* was cultivated in a mineral salt medium, as described by Schulze et al. (2014), containing a phosphate buffer system (8.99 g/L KH_2_PO_4_ and 0.12 g/L Na_2_HPO_4_ × 2 H_2_O), 0.1 g/L sodium citrate × 2 H_2_O, 0.1 g/L yeast extract, 0.2 g/L MgSO_4_ × 7 H_2_O, 4.72 g/L (NH_4_)_2_SO_4_. After autoclaving 2% (v/v) of sterile trace elements solution with 4 g/L CaCl_2_ × 2 H_2_O, 0.55 g/L FeSO_4_ × 7 H_2_O, 0.475 g/L citric acid, 0.1 g/L ZnSO_4_ × 7 H_2_O, 0.076 g/L MnSO_4_ × H_2_O, 100 μL/L 18 M H_2_SO_4_ and 2% (v/v) of sterile salts solution comprising 20 g/L MgSO_4_ × 7 H_2_O, 10 g/L yeast extract were added. Additionally, glucose was supplemented aseptically at concentration of 50 g/L. The yeast cells were activated in pre-cultures in conical shake flasks by scratching yeast cells from YM agar (3 g/L yeast extract, 3 g/L malt extract, 5 g/L peptone, 20 g/L agar, pH 7, sterily supplemented with 10 g/L glucose after autoclaving). The first pre-culture was performed in 50 mL medium at 130 rpm and 20°C for 24 h. Second pre-culture was inoculated from first pre-culture to an OD_600nm_ of 1.0 in 200 mL medium and cultivated at the same parameters for 24 h. Lipid production was conducted in duplicates in a 2.5 L bioreactor (Infors HT, Bottmingen, Switzerland; Minifors fermentor) with 1.2 L mineral salt medium with initial OD_600nm_ of 1.0. The cultivation was performed at pH 4 (automatically controlled by addition of 4 M H_3_PO_4_ and 4 M NaOH), 22.5°C, 600 rpm and with 1 vvm aeration rate. To prevent foam formation, the bioreactors were equipped with a foam probe (Infors HT, Bottmingen, Switzerland) using Contraspum A 4050 HAC (Zschimmer und Schwarz GmbH und Co KG, Lahnstein, Germany) as an anti-foaming agent. Every 24 h a manual feed of 2% (v/v) sterile trace elements solution, 2% (v/v) sterile salts solution and glucose supply to 55 g/L was implemented after determining the consumed carbon amount. The yeasts growth was monitored by daily sampling and analysis of OD_600nm_, CDW, glucose and ammonium consumption. SCO production was indirectly analyzed by gas chromatography via acidic direct transesterification as described in Gorte et al. (2020). After 6 days the biomass was harvested and directly further processed.

### Glucose Determination

Glucose consumption was determined enzymatically from sample supernatant using the UV-method at 340 nm of the D-glucose test kit from R-Biopharm (Art. No. 10716251035, R-Biopharm AG, Darmstadt, Germany). The method was performed according to the manufacturer’s instructions, but all volumes were reduced to one-third.

### Ammonia Quantification

Ammonium nitrogen in sample supernatant was analyzed photometrically by use of the Spectroquant kit (1.14752.0001, Merck KGaA, Darmstadt, Germany). All volumes were down scaled to 300 μL per sample and measured in microtitre plates in duplicates according to the manufacturer’s instructions.

### Cell Dry Weight

The determination of CDW was performed in triplicates. For each sample, 1 mL of culture broth was added in a reaction tube and centrifuged at 6000 × *g* for 5 min. The cell pellet was washed with sterile physiological saline (0.9% w/v NaCl) and resuspended in 1 mL saline. The yeast suspension was poured into a pre-weighted (DW_empty_ [g]) aluminum cap. The exact masses of 1 mL yeast suspension (*w*_suspension_ [g]) and, separately, of 1 mL pure saline (*w*_saline_ [g]) were determined using a precision balance. All caps were dried in a drying oven at 90°C for about 24 h. Afterward, the weight of the dry biomass and saline was measured again (DW_full_[g]). CDW was calculated using Eq. (1).

(1) $$C\,D\,W\,\left[\frac{g}{k\,g_{s\,u\,s}}\right]=\left(\frac{\begin{array}{c} (DW_{f\,u\,l\,l}-\\ DW_{e\,m\,p\,t\,y}){}_{s\,u\,s\,p\,e\,n\,s\,i\,o\,n} \end{array}}{w_{s\,u\,s\,p\,e\,n\,s\,i\,o\,n}}-\frac{\begin{array}{c} (DW_{f\,u\,l\,l}-\\ DW_{e\,m\,p\,t\,y}){}_{s\,a\,l\,i\,n\,e} \end{array}}{w_{s\,a\,l\,i\,n\,e}}\right)\times \,1000$$

### Lipid Extraction

The lipid extraction protocol was adapted from the one developed for microalgae and fully detailed in Silve et al. (2018b). In brief, 15 mL of the yeast suspension at approximately 20 g_DW_/L were centrifuged, supernatant was discarded and the wet biomass pellet was resuspended in 16 mL of 100% ethanol and 6,5 mL of hexane in order to reach a final extraction system of water/ethanol/hexane, 1:18:7.3 vol/vol/vol. Note that the water in the extraction system corresponds to the remaining water in the yeast pellet, i.e., no water was intentionally added. Extraction took place in Teflon tubes (Nalgene Oak Ridge Centrifuge Tubes, Teflon FEP, 50 mL Thermo Scientific), overnight, with agitation and in the dark. After 20 h, the Teflon tubes were centrifuged and 10 mL of the solvent containing the raw extract were pipetted and complemented with 30 mL of hexane and 5 mL of water in order to accomplish phase separation. The upper hexane phase was collected, and evaporated under nitrogen flow. Extraction yields were determined gravimetrically with a precision balance.

### Yeast Composition

#### Sample Preparation for Composition Analysis

Composition analysis was performed with a 0.02% (w/v) saline washed yeast suspension. The washed yeast suspension was centrifuged at 10,000 × *g* for 10 min and supernatant was discarded. The pellet was frozen at −20°C before subsequent freeze-drying for 24 h in a laboratory freeze-drier (Alpha 1–4 LDplus, Christ). The freeze-dried pellet was then kept at −20°C before further analysis of content.

#### Total Lipid

Total lipid extraction was performed with a commercial Soxhlet apparatus (behrotest Kompakt-Apparatur KEX 30 from Behr Labor-Technik). Approximately 0.5 g of the washed and freeze-dried yeast biomass was precisely weighted and bead-milled for 5 min at 30 Hz in stainless steel cups (Grinding jar for MM 400, 50 mL, 01.462.0216, Retsch, Haan, Germany) using nine 12 mm stainless steel beads (05.368.0037, Retsch) and a commercial bead-miller (Mixer mill, MM400, Retsch). The biomass was placed inside a permeable paper thimble (Extraction Thimbles Cellulose, 90022080, Albet LabScience, Dassel, Germany) and deposited inside the Soxhlet chamber. Approximately 50 mL of hexane was used with a heating temperature of 170–200°C. The extraction was run for at least 3 h, which corresponded to at least 20 extraction cycles. At the end of the extraction, the solvent was siphoned out of the apparatus and the boiling flask along with extracted lipids was removed, let to cool down under nitrogen atmosphere, and the total lipid content was determined gravimetrically.

Note that Bligh and Dyer as well as Folch protocols were also tested as described in our previous work (Gorte et al., 2020) but gave lower estimations of the total lipid content.

#### Total Protein

Total protein content of the yeast biomass was evaluated using sodium hydroxide extraction at high temperature. Approximately 10 mg of washed and freeze-dried yeast powder was resuspended in 2 ml of sodium hydroxide (1 M) and incubated at 95°C for 1 h. After this incubation, samples were cooled to ambient temperature, centrifuged at 4500 ×*g* for 10 min, and the supernatant was processed for protein determination applying a modified Lowry method (DC Protein Assay, BioRad), using bovine serum albumin as standard.

#### Total Carbohydrates

Determination of carbohydrate was performed using the Anthrone Sulfuric Acid assay. Fresh starch aqueous solutions with concentrations ranging from 0.02 to 0.2 g/L were prepared from starch powder (Merck 1.01257). They were used as standards and processed like the samples. Approximately 10 mg of the washed and freeze-dried yeast biomass was precisely weighted and diluted in distilled water to an exact concentration of 1 mg/mL. All samples were processed in duplicates. The anthrone reagent was prepared on the day of the experiment by dissolving anthrone (Merk 1.01468) in 95% sulfuric acid (AnalaR NORMAPUR: VWR Chemicals 20700) at a final concentration of 0.1% (w/v). The solution was well mixed and kept on ice for at least 5 min. Afterward 400 μL of diluted sample or standard were transferred into 1.5 mL Eppendorf Safe Lock tube. 800 μL of anthrone reagent were added and homogenized with the sample solution through inversion. After 5 min of incubation on ice, the mixed solution was transferred into a thermo-incubator, pre-heated at 95°C and shaken at 300 rpm for 16 min, and then cooled down on ice. Optical density of the cooled samples was measured in triplicate at 625 nm and carbohydrate concentration was calculated using the standard curve and by considering the dilution factors.

#### Total Inorganic Content

Approximately 200 mg of washed and freeze-dried yeast biomass was measured on a precision balance and heated for 20 h at 650°C in a high temperature furnace (Hochtemperaturofen Supertherm HT04/17, Nabertherm, Germany). After removal from the furnace, the samples were let to cool down to room temperature, whereupon they were measured again on a precision balance allowing for the calculation of the inorganic solid content designated in the paper as ashes content.

### Yo-Pro Staining

Yo-pro staining is generally used as a marker of membrane integrity. In particular, it was used in this study to detect how washing could render cells permeable. Permeabilized cells will appear positive to Yo-Pro staining. The initial yeast samples at approximately 20 g/L were diluted 1:300 with their own medium filtered at 0.2 μm. 1 mL of the sample was supplemented with 10 μL of Yo-Pro (YO-PRO-1 Iodide (491/509), Invitrogen, Thermo Fisher Scientific) at 0.1 mM. The sample was left 10 min at room-temperature for staining and then diluted 1:5 before input in the flow cytometer. Flow cytometer measurements were conducted on an Attune NxT (Thermo Fisher Scientific) with a 488 nm laser as excitation source. Emission fluorescence signal was collected with the green filter of the device (530/30).

### PEF Treatment

**FIGURE 1:**
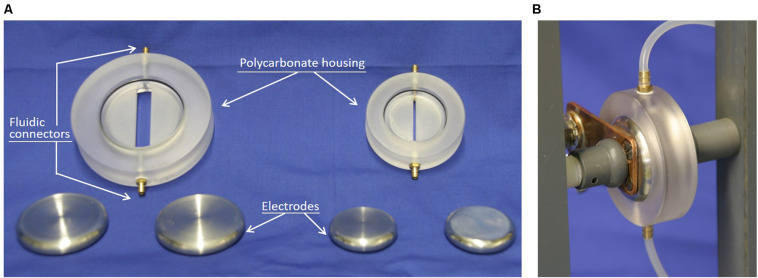
Photos of the PEF-treatment chambers. **(A)** Disassembled treatment chambers. The chambers are designed for treating suspensions with conductivities of 1.5 mS/cm (left) and 12 mS/cm (right) with a 50 Ohm transmission-line generator. **(B)** Illustration of an assembled treatment chamber with the tubes used for the continuous flow of the yeast suspension.

The yeasts were treated either directly after harvesting or after a washing step, i.e., in suspension with a conductivity at 20°C of either 10.14 ± 0.63 or 1.27 ± 0.09 mS/cm. In both cases, PEF-treatment was performed in continuous flow. Two treatment chambers were used, one for each of the two conductivities mentioned above. They consisted of two parallel circular stainless-steel electrodes separated by a polycarbonate housing (Figure 1) imposing a distance between the electrodes of 4 mm. In order to deliver square shaped pulses, the setup needs to ensure impedance matching, i.e., the output impedance of the generator (50 Ω in our case) needs to match the impedance of the load, which consists in the treatment chamber filled with the suspension to be treated. Therefore, the dimensions of the volume between the electrodes filled with suspension to be treated were chosen to ensure that the impedance of the chambers is equal to 50 Ω when chambers were filled with suspensions with a conductivity of 1.5 and 12 mS/cm, respectively. Considering the increase in conductivity caused by the temperature increase that occurs during the treatment inside the chamber, the treatment chambers are ideal to treat suspension with initial conductivities of 1–1.2 and 8–10 mS/cm, respectively, and therefore suitable for processing washed and unwashed yeast suspensions. Please note, that more detailed explanations on impedance matching or impedance computation in general, can be found in the appropriate textbooks (Smith, 2002) or publications (Silve et al., 2012).

PEF treatment was performed with a custom-made transmission-line generator with an output impedance of 50 Ω. Pulse duration, was fixed at Δ*t* = 1 μs and electric field intensity was varied between *E* = 1.4 and *E* = 4 MV/m. The electric pulses were applied continuously with a pulsing repetition rate *f*_rep_ adjusted between 0.1 and 12 Hz, in order to adjust the specific treatment energy. The flow rate of the yeast suspension inside the treatment chambers was fixed at 0.1 mL/s except in some specific cases (mentioned along in the manuscript) for which it was reduced to 0.05 mL/s in order to apply the required specific energies.

### Conductivity Measurement

The conductivity σ [μS/cm] of the yeast suspensions was measured using a conductivity meter (Endress + Hauser, CLM 381). No automatic temperature compensation was used, but temperature *T* [°C] was recorded in parallel with conductivity. The equivalent conductivity at 20°C, σ_20_ [μS/cm], was calculated using Eq. (2) where α_20_ is the temperature coefficient of variation at 20°C (Grimnes and Martinsen, 2008). The coefficient α_20_ was obtained experimentally by measuring conductivity of a yeast suspension at different temperatures (data not shown) and had a value of 2.58%/°C.

(2) $$\sigma_{20}=\sigma_{T}\,\frac{1}{1+\alpha_{20}\,\left(T-20\right)}$$

### Statistical Analysis

Results reported in the manuscript were obtained from n independent experiments (*n* = 2 or 3) with internal duplicates in each experiment. Duplicate values were averaged for one output value per experiment. These output values were used to calculate averages and standard deviations (std) on n samples displayed in the graphs. Statistical significance was evaluated using the unpaired *t*-test. *P*-values less than 0.05 are indicated with ^∗^, less than 0.01 with ^∗∗^ and less than 0.001 with ^∗∗∗^.

## Results

### Yeast Cultivation

**FIGURE 2:**
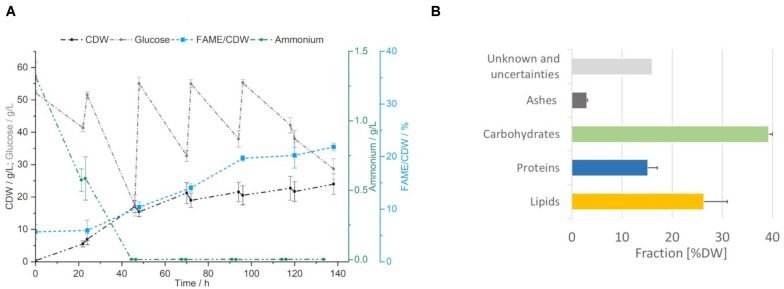
Yeast cultivation. **(A)** Growth curve of *S. podzolica* on mineral salt medium and daily fed glucose, ammonium consumption, biomass (CDW) and lipid production (indirectly as FAME/CDW) are indicated as average ± standard deviation of four independent cultivations. **(B)** Composition of the yeast on harvesting day. Average + standard deviation of four independent cultivations.

To produce fresh yeast biomass for the PEF treatment, *S. podzolica* was cultivated in 2.5 L bioreactors with constant parameters at 22.5°C, pH 4, aeration rate of 1 vvm and 600 rpm for 6 days. In Figure 2A the cultivation curve is presented, highlighting the most important constants. For SCO production the excess of carbon is essential and was assured by a daily supplement of glucose to the concentration of 55 g/L. The last glucose feed was performed at 96 h. Ammonium, as nitrogen source, was depleted after the second cultivation day, resulting in 64.4% of the total yeast biomass. Within the next days the production of the biomass flattened due to limited nitrogen replenishment in form of yeast extract in the daily fed salts solution. The maximum biomass concentration was reached at the end of the cultivation at 138 h with about 20 g/L CDW. Upon nitrogen limitation the lipid production was induced. For analytical purposes, the transesterifiable lipids were daily tracked as fatty acid methyl esters per CDW (% FAME/CDW), which enabled to observe constant increase in the FAME content with the highest value of 21.8 ± 0.7% FAME/CDW reached after 138 h.

Composition of the yeasts on the harvesting day was analyzed and is reported in Figure 2B. Biomass composition showed good reproducibility and in total more than 86% of the biomass could be attributed to the following fractions: lipids 26.4 ± 4.6%, proteins 15.2 ± 1.9%, carbohydrates 39.3 ± 0.7% and ashes 3.1 ± 0.1%. A systematic fraction of 16% could not be identified and was missing to close the mass balance. This non- identified fraction is suspected to be some complex molecules of the resistant cell wall but further investigations were not carried out in the course of this study.

### Unwashed and Washed Routes

When performing PEF-treatment in a continuous flow manner, the specific energy of the treatment W [J/kg_DW_] can be expressed using Eq. (3), where E [V/m] is the electric field, Δ*t* [s] is the duration of the pulses, *f*_rep_ [Hz] the pulse repetition rate, *t*_res_ [s] the residency time of suspension inside the treatment chamber, C [kg/m^3^] the biomass concentration and σ [S/m] the conductivity of the suspension inside the treatment chamber. Treatment specific energy, therefore, depends on the treatment parameters, which should be chosen based on their efficiency (Escoffre et al., 2009), but also on the properties of the suspension to handle, especially its concentration and conductivity.

(3) $$W=E^{2}\times \sigma \times t_{\mathrm{res}}\times \frac{\Delta \,t\times f_{\text{rep}}}{C}$$

**FIGURE 3:**
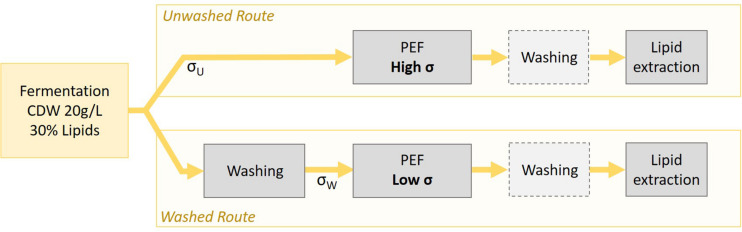
Schematic of the two treatment paths “Unwashed” and “Washed”.

Yeast suspension collected from the bioreactor at the end of a cultivation cycle had an average CDW of 20.2 ± 2.9 g/L (i.e., a concentration of 20.2 ± 2.9 kg/m^3^). This was kept as a working concentration for all experiments in this study. Regarding the conductivity of the suspension, the value of unwashed suspension directly harvested from the reactor was σ_U_ = 10.14 ± 0.63 mS/cm at 20°C. A suspension with such a conductivity can in principle be directly treated but at the expense of a high-energy consumption. To avoid the high-energy cost of PEF, an alternative is to wash the yeast suspension although this also represents energy cost, as well as fresh water consumption. The present study therefore investigated two different processing routes as described in Figure 3: the “unwashed route” in which PEF-treatment is applied on the yeast suspension directly after harvesting and the “washed route” in which the yeast suspension is washed before the PEF-treatment. In both cases, the impact of a supplementary washing step after the PEF-treatment and before the subsequent lipid extraction, was tested.

The washing procedure was performed in order to reduce the initial conductivity of the suspension σ_U_ by a factor 8, in order to reach a value σ_W_ close to 1.2 mS/cm. The two following washing procedures were tested for their impact on cell permeabilization and on further lipid extraction.

(A) Add distilled water to the yeast suspension until the desired conductivity (i.e., eight times lower than unwashed suspension) is reached. Centrifuge and remove the excess supernatant to recover the initial cell concentration.
(B) Centrifuge the suspension and remove the supernatant. Add then the same amount of distilled water and enough NaCl to reach the desired conductivity (i.e., 8 times lower than unwashed suspension).

**FIGURE 4:**
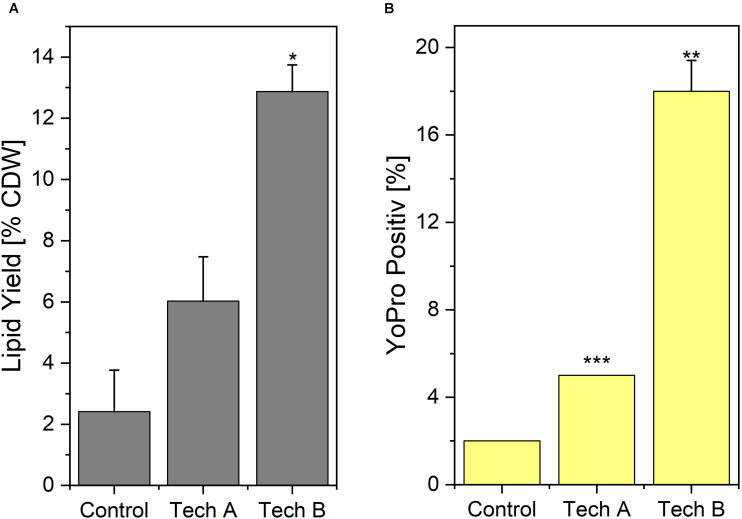
Impact of washing on lipid extraction **(A)** and Yo-Pro uptake **(B)**. Two different washing techniques were implemented. In technique A, distilled water was added to reduce conductivity in yeast broth prior to centrifugation and in technique B, the yeast suspension was centrifuged beforehand, subsequently the biomass was resuspended to the same concentration as before with distilled water supplemented with NaCl to reach the targeted conductivity. Results are average ± standard deviation of two independent experiments (*n* = 2) with duplicates in each. Control refer to yeast processed directly after harvest i.e., not submitted to any washing treatment. Statistical significances are indicated in comparison with control.

Results of Yo-Pro staining and lipid extraction yield obtained after washing using the two above-mentioned techniques, are displayed on Figure 4. With washing technique A, the percentage of Yo-pro positive cells i.e., permeable cells, remained low i.e., only 5% compared to 2% for the absolute unwashed control. This percentage reached 18% using washing technique B. Regarding lipid extraction using ethanol-hexane blend, the yields normalized to the CDW were 2.41% for the unwashed control and 6.0 and 12.9%, for the yeasts washed with technique A and technique B, respectively.

Based on those experiments, the washing technique A appeared as less harmful to the yeast. Washing technique B induced more damage but this method required less water and the volumes to be centrifuged were almost ten times lower. For those practical reasons, which are crucial at an industrial level, washing technique B was chosen for the rest of the study. The average conductivity obtained after the washing procedure in the rest of the study, i.e., during the lipid extraction experiments, was σ_W_ = 1.27 ± 0.09 mS/cm at 20°C

### Lipid Extraction After PEF Treatment in the Unwashed Route

**FIGURE 5:**
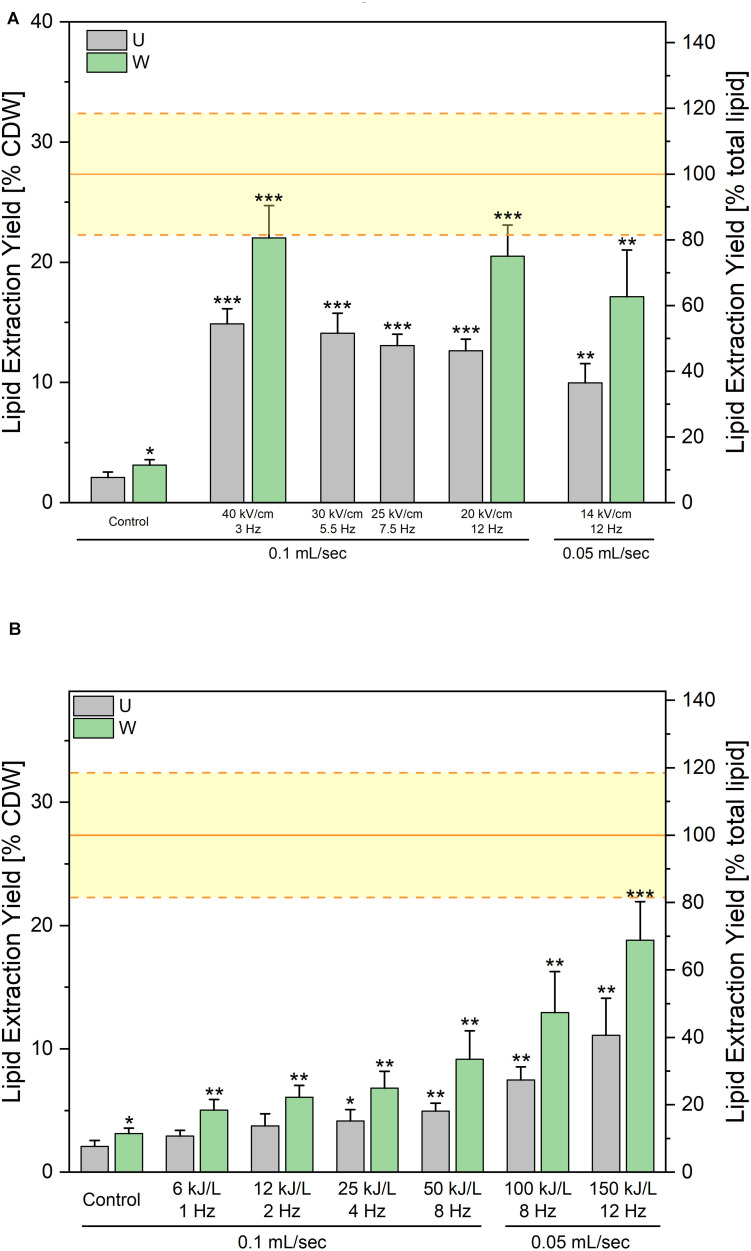
Lipid extraction yields obtained after processing along the “unwashed-route”. PEF-treatment was applied directly after harvesting. Pulse duration was kept constant at Δ*t* = 1 μs. **(A)** Impact of the electric field value at a constant specific treatment energy of 150 kJ/L. The *x*-axis indicates for each condition the applied electric field in kV/cm (upper values), the repetition rate in Hz at which pulses were applied (middle values) and the working flow rate of the suspension in the treatment chambers (lower values). **(B)** Impact of the specific treatment energy at a constant electric field value of 14 kV/cm. The applied specific energies, the repetition rate in Hz at which pulses were applied as well as the working flow rates have been indicated on the x-axis. For both graphics, the gray bars are the results of lipid extraction performed directly after the PEF-treatment (U) and the green bars are the lipid extraction yields obtained when a washing step is added after PEF-treatment before addition of extraction solvents (W). The yellow lines indicate the average ± standard deviation of Soxhlet extractions, as absolute control. Results are reported as the average + standard deviation of three independent experiments (*n* = 3) with samples processed in duplicates. Statistical significances are indicated in comparison with the unwashed control.

First experiments conducted on unwashed yeast suspension intended to screen pulse parameters. Pulse duration was not varied in this study and was kept at 1 μs. The electric field magnitude was varied from 14 to 40 kV/cm and the repetition rate was adjusted in order to keep an applied energy of 150 kJ per liter of suspension in all cases. Results are displayed on Figure 5A. As can be seen, the lipid extraction yield obtained after the solvent extraction was 15% when electric field magnitude was fixed at 40 kV/cm. By decreasing the electric field magnitude at constant specific energy, the extraction efficiencies were slightly reduced, reaching 10% at 14 kV/cm. For a few conditions, a washing step was added after the PEF treatment just before lipid extraction. This extra washing step increased all yields, enabling to reach up to 22% of CDW. Nevertheless, in the unwashed route, none of the tested PEF-treatment conditions enabled to extract more than 54% of the evaluated lipid content when no washing was added after PEF-treatment. This value increased to 81% in case a washing step was added, leaving therefore 19% of lipids unextracted.

In a next step, the impact of energy input was tested, keeping the electric field value at 14 kV/cm. For that matter the repetition rate and therefore the average number of pulses applied, was varied in order to test specific applied energies ranging between 6 and 150 kJ/L. Results are presented on Figure 5B and show that already the lowest tested energy i.e., 6 kJ/L positively impacts the lipid extraction yield, although with too low extraction efficiency. Extraction yield then gradually increased with the applied energy, with the maximum extraction efficiency obtained for the maximum tested energy i.e., 150 kJ/L. For all tested energies, extraction yields significantly increased when yeast suspension was washed after the PEF-treatment, before proceeding with the lipid extraction.

### Lipid Extraction in the Washed Route

**FIGURE 6:**
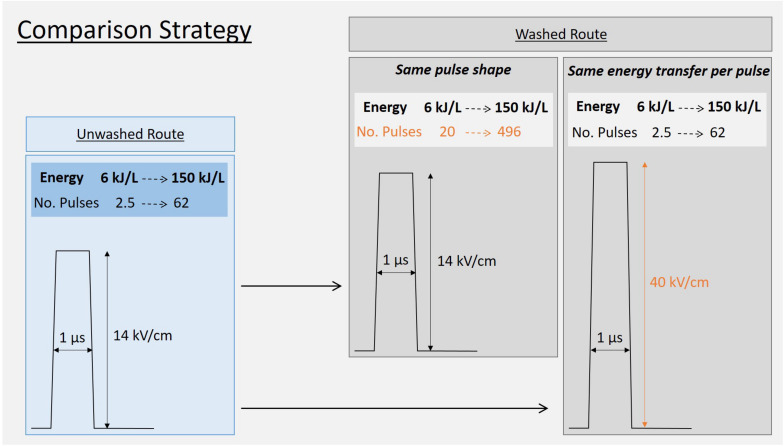
Strategy to compare “washed-route” with the “unwashed-route.” In the washed route the conductivity of the yeast suspension is 8 times lower than in the “unwashed-route”.

In order to compare the washed route with the previously tested unwashed route, two experiments were designed as sketched in Figure 6. The strategy consisted in mirroring the experiment described above (Figure 5B), i.e., testing the efficiency of specific energies ranging from 6 to 150 kJ/L either by,

(I) Exact application of the same pulse shape, i.e., *E* = 14 kV/cm and Δ*t* = 1 μs. This implied the application of more pulses in order to compensate for the lower conductivity of the washed yeast suspension and to achieve the targeted specific energies.

or by

(II) Transfering the same energy per pulse into the suspension, which implied increasing the electric field strength to 40 kV/cm to compensate energy transfer for the lower conductivity of the washed yeast suspension.

**FIGURE 7:**
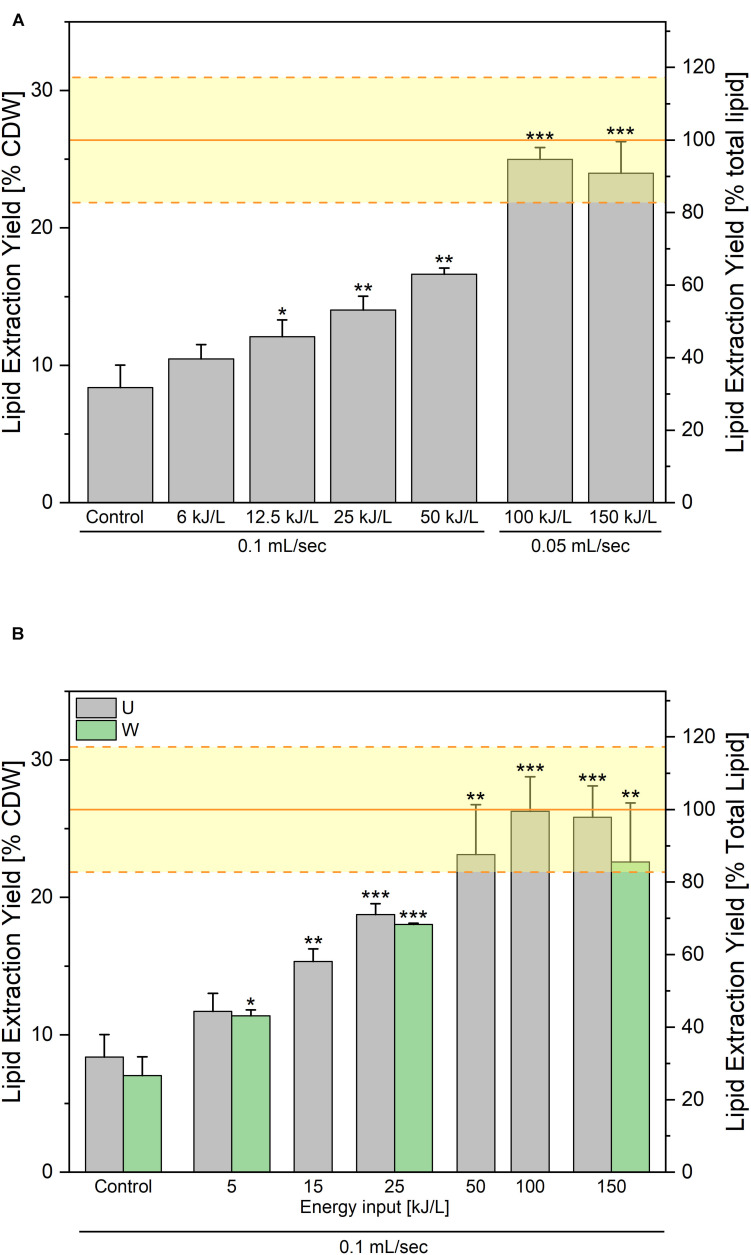
Lipid extraction yields obtained after processing along the “washed-route.” PEF-treatment was applied on the yeast washed after harvesting. Impact of the specific treatment energy at a constant electric field value of **(A)** 14 kV/cm and **(B)** 40 kV/cm. For the bottom graph, the gray bars are the results of lipid extraction performed directly after the PEF-treatment and the green bars are the lipid extraction yields obtained when a washing step is added after PEF-treatment before addition of extraction solvents. The yellow lines illustrate the average ± standard deviation of Soxhlet extractions, as absolute control. For both experiments, the results are reported as the average + standard deviation of three independent experiments (*n* = 3) with samples processed in duplicates. Statistical significances are indicated in comparison with the unwashed control.

The results of the two experiments are displayed in Figure 7. In both cases an increase of the lipid extraction yield is observed with increasing PEF treatment energy input. In case the electric field is fixed at 14 kV/cm (Figure 7A), 95% of the lipid content (25% of CDW) can be extracted with a specific energy of 100 kJ/L. Further increasing the energy to 150 kJ/L did not further improve the extraction yield. In case the electric field was increased to 40 kV/cm (Figure 7B), even higher yields were obtained, with already 87% of the lipid recovered at a specific energy of 50 kJ/L and up to 99% at 100 kJ/L. Note that in that case, the impact of an additional washing step between the PEF-treatment and the lipid extraction was tested, and that it appeared as not providing any significant improvement of the lipid recovery.

## Discussion

The present study explores the possibility to use PEF-treatment as a pre-treatment method to weaken oleaginous yeast cells prior to solvent extraction. The study was performed on the recently characterized yeast *Saitozyma podzolica*, for which mechanical pre-treatment such as bead-milling and high pressure homogenization were efficient only at extremely high energy inputs (Gorte et al., 2020). In order to evaluate the benefit of PEF-pre-treatment for lipid extraction, yeast suspension was either processed directly after harvesting (unwashed route) or after a washing step aiming at reducing the conductivity (washed route). In the unwashed route, the best lipid extraction yields were obtained using pulses of 1 μs with an electric field strength of 40 kV/cm and an energy of 150 kJ/L of suspension (see Table 1). The extraction efficiency was, however, only 15% reported to CDW i.e., about 50% of the total lipid content evaluated by Soxhlet. In case a washing step was added after the PEF-treatment, just before the lipid-extraction step, the final yield slightly increased and up to 81% of the total lipid were recovered. Much better results were obtained in the washed route i.e., when treating the lower-conductivity yeast suspension. In that case, 87 and 99% of the evaluated lipid content were recovered with energy input of 50 and 100 kJ/L, respectively (Table 1).

**TABLE 1:** Energies involved for different treatment conditions chosen based on their efficiency.

Route	PEF parameters	Energy [kJ/L]	Energy [kJ/kgDW]	Lipid extraction yield [% Lipid per CDW]	Lipid extraction yield [% Lipid per Lipid content]	Energy [kJ/kg lipid]	Energy [kWh/kg lipid]
Unwashed-route CDW = 20.21 ± 2.87	1 μs 40 kV/cm	150	7 422	15	54	49 480	13.74
				22	81	33 737	9.37
Washed-route CDW = 19.96 ± 3.22	1 μs 14 kV/cm	100	5 010	25	95	20 040	5.57
	1 μs 40 kV/cm	50	**2 505**	**23**	**87**	**10 891**	**3.03**
	1 μs 40 kV/cm	100	5 010	26	99	19 269	5.35

*The values underlined correspond to values obtained after addition of a washing step after PEF-treatment before lipid extraction. Bold values indicate the best obtained results.*

The reason for a higher efficiency of the washed route has not be further investigated but several hypotheses can be proposed. First, it might be that the whole procedure i.e., PEF-treatment and lipid extraction, is more efficient on washed yeast biomass since cells are already weakened by washing (Figure 4). However, since a washing step added in the “unwashed-route” immediately after PEF-treatment (i.e., before the lipid extraction) helped to recover higher yields, a second hypothesis is that washing is not required for the PEF treatment itself but more for the extraction. Some constituents of the medium, such as the high glucose content, might indeed interact with the solvents and reduce the extraction efficiency. In order to test this hypothesis, a possibility would be to reduce the number of possible elements disturbing the extraction at the end of cultivation by improving the medium composition and especially by stopping the sugar feeding at the end of cultivation in order not to have any sugar in excess. Since this optimization of medium is required in any case, to reduce the costs of up-stream related to nutrients, it appears as an obvious next step to this work. Finally, the higher efficiency of the “washed-route” might be simply explained by the fact that at identical energy input, the PEF-parameters used in the “washed-route” are harsher either in terms of number of pulses or of field intensity. Indeed, the fact that conductivity is 8 times lower in the “washed-route” enables for given pulse parameters to apply 8 times more pulses or for a given number of pulses to apply an electric field $\sqrt{8}$ (i.e., ∼ 2.8) times higher. In any case, despite the fact that this study demonstrates that PEF-treatment can be applied directly after the harvest of the yeast, it appears that in practice a washing step will still be required for the current way of cultivation. Future cultivation optimization efforts should go for reducing the nutrients content in suspension at the time of harvest.

Regarding the absolute energy consumption, the best parameters of the study enabled to recover lipid using 10 891 kJ/kg_LIPID_ (Table 1) i.e., 3 kWh/kg_LIPID_. To classify this energy balance with studies of other oleaginous yeasts, it is evident that our method is similarly competitive in terms of energy consumption compared to reported low energy demanding methods. As reviewed by Dong et al. (2016) for the hydrochloric acid digestion under heat of *Cryptococcus curvatus* biomass 9.3–18.6 MJ/kg_LIPID_ (2.58–5.17 kWh/kg_LIPID_) is required (Yu et al., 2015), for the enzymatic digestion of *Rhodosporidium toruloides* biomass the energy consumption of 13.3 MJ/kg_LIPID_ (3.67 kWh/kg_LIPID_) is reported (Jin et al., 2012). However, cell lysis by acidic hydrolysis is disadvantageous due to formation of corrosion in the process equipment and the use of enzymes for cell wall digestion causes high costs on large scale. In contrast, mechanical methods, such as bead milling, are considered to be highly scalable but are more energy demanding. For the oleaginous yeast *Yarrowia lipolytica* bead milling, as one of the most energy efficient pre-treatment methods for lipid extraction, consumed 115.2 MJ/kg_LIPID_ (32 kWh/kg_LIPID_) of energy (Meullemiestre et al., 2016), which is about 10-fold higher than the other discussed methods including our presented PEF method.

For the PEF method the amount of energy in this current study can most probably be further reduced using standard strategies related to PEF-treatment. First of all, PEF-parameters can be improved since they have crucial role in PEF efficiency (Escoffre et al., 2009). The importance of the parameters can be seen already on the fact that in the “washed-route”, for a constant energy input of 100 kJ/L, pulses of 40 kV/cm were more efficient than pulses of 14 kV/cm (Table 1). Further improvements should therefore be made for example by optimizing pulse duration. Additionally, it is possible to reduce the energy consumption by treating a yeast suspension with higher cell concentration since PEF-treatment efficiency does not decrease for denser cell suspension (Goettel et al., 2013). Required PEF-treatment energy linearly decreases with increasing biomass density in the suspension to be treated (Goettel et al., 2013). Only limitation for this approach will be the viscosity of the suspension, which should not increase too much, in order not to increase energy requirement of pumping during the continuous flow process. Finally, an incubation period could be added after PEF-treatment since it was shown in the past for microalgae that this strategy allowed reducing energy input up to 6 times (Silve et al., 2018a).

The approach presented in this study was developed sticking as much as possible to the requirements of industry in order to offer a downstream approach that can be up-scaled. In particular, no freezing or freeze-drying was used, since those two processes are known to be highly energy-demanding (Willis et al., 2014). Apart from the possible improvements regarding the energy demand of the PEF-treatment, additional efforts in the future should be focused on reducing the amount of solvent and also switching to more sustainable ones. Currently, most existing studies agree that a system of polar and non-polar solvents is the most efficient approach to recover all lipids. For analytical purposes the most popular and effective methods at laboratory-scale are systems using chloroform and methanol according to Folch (Folch et al., 1957) or Bligh and Dyer (Bligh and Dyer, 1959). However, chloroform is highly toxic and carcinogenic and its usage should be avoided even in the laboratory (Prat et al., 2015). The ethanol-hexane blend used in this study is for that matter more suitable. Indeed, for potential industrial scale application hexane is considered as a less toxic alternative for chloroform and it is already commonly used in food industry (Biondo et al., 2015). However, hexane is still a petro-based solvent and, therefore, from environmental point of view not reasonable. Further work should, therefore, focus specifically on the choice of solvent in order to propose a solvent system both economically viable and sustainable. Not only the type of solvent but also the volumes at play and the easiness to recycle will be determinant for the implementation at larger scale.

In future studies, we aim to examine in detail the energy and economic costs associated with the whole process, from cultivation of the yeast until lipid extraction. Such an analysis will enable to propose a realistic evaluation of the potential applications for the lipids extracted using our processing route.

## Conclusion

To satisfy the increasing energy and resources demand for our growing population in the next decades, research and finding of sustainable realization procedures are urgently needed. Biotechnologically produced microbial oils could play an important role in this respect and be potential alternatives for crude and plant oils. In this study we demonstrate on fresh biomass from the oleaginous yeast *Saitozyma podzolica* DSM 27192, that lipid extraction efficiencies are remarkably enhanced by PEF-treatment of the biomass prior to organic solvent lipid recovery. Moreover, by applying a washing step before PEF-treatment the extraction yield increased to 99% of total lipid. We show that the amount of required energy is reasonable and competitive with other low-energy consuming yeast pre-treatment methods, such as enzymatic digestion. This study is the first insight in the PEF technique of oleaginous yeasts. With further optimization toward feasibility of up-scaling the potential for industrial implementation will be increased.

## Data Availability Statement

The raw data supporting the conclusions of this article will be made available by the authors, without undue reservation.

## Author Contributions

OG performed yeast cultivation. OG, NN, IP, and AS performed the PEF experiments and lipid extractions and assisted by RW and KL who operated PEF devices. AS, OG, and NN designed and planned the study and analyzed the data. AS and OG drafted the manuscript. KO, WF, and CS constructively contributed to the content. All authors critically revised the article.

## Conflict of Interest

The authors declare that the research was conducted in the absence of any commercial or financial relationships that could be construed as a potential conflict of interest.
